# Genomic epidemiology and antimicrobial resistance of *Morganella* clinical isolates between 2016 and 2023

**DOI:** 10.3389/fcimb.2024.1464736

**Published:** 2025-01-31

**Authors:** Wentao Zhu, Qian Liu, Jinlv Liu, Yaqi Wang, Hong Shen, Ming Wei, Ji Pu, Li Gu, Jing Yang

**Affiliations:** ^1^ Department of Infectious Diseases and Clinical Microbiology, Beijing Institute of Respiratory Medicine and Beijing Chao-Yang Hospital, Capital Medical University, Beijing, China; ^2^ Department of Clinical Laboratory, Beijing Anzhen Hospital, Capital Medical University, Beijing, China; ^3^ National Key Laboratory of Intelligent Tracking and Forecasting for Infectious Diseases, National Institute for Communicable Disease Control and Prevention, Chinese Center for Disease Control and Prevention, Beijing, China; ^4^ Academy of Medical Sciences, Shanxi Medical University, Taiyuan, China; ^5^ Hebei Key Laboratory of Intractable Pathogens, Shijiazhuang Center for Disease Control and Prevention, Shijiazhuang, Hebei, China

**Keywords:** *Morganella morganii*, antimicrobial resistance, clinical, whole-genome sequencing, taxonomy

## Abstract

*Morganella morganii* is a Gram-negative, opportunistic pathogen that is often associated with nosocomial infections. Here, the genomic characteristics and antimicrobial resistance (AMR) of *Morganella* clinical isolates between 2016 and 2023 were determined. A total of 218 clinical isolates were mainly identified from urinary tract (48.2%) and respiratory tract (16.5%), with 105 isolates randomly selected for whole genome sequencing. The highest rates of antibiotic resistance were observed with SAM (68.3%), followed by CIP (39.9%), and SXT (37.2%). Distance analysis suggested that the 105 newly sequenced isolates could be divided into two groups: *M. morganii* subsp. *morganii* and *M. morganii* subsp. *sibonii*. While, the average nucleotide identity between these groups showed only 91.5-92.2% similarity, raising the possibility that they may be distinct species. Phylogenomic analysis revealed that the 102 *M. morganii* isolates fell into six clades, with clades 4-6 making up the majority. Core genome multi-locus sequence type analysis indicted high genomic diversity among different hosts and relatively stability (< 10 SNPs accumulated over three years) within the same host. Together with epidemiological data, isolates of four genetic clusters could be possible nosocomial transmissions. The identified 80 AMR genes belonged to 15 drug-related classes, with *tet(B)* gene being the most prevalent, followed by *sul1*, *catA2*, and *sul2* genes. This study provided comprehensive genomic insights and AMR patterns of *Morganella* isolates in China, highlighting the necessity for continuous monitoring through whole genome sequencing.

## Introduction

*Morganella morganii* (*M. morganii*), belonging to the family *Morganellaceae* of the order Enterobacterales, is a Gram-negative, facultatively anaerobic, rod-shaped bacterium (Adeolu et al., 2016). It was firstly reported as *Proteus morganii* in 1906 by Morgan et al. from children with summer infantile diarrhea (O’Hara et al., 2000). Later, in 1943, Fulton classified these strains into the genus *Morganella*, which included two species: *M. morganii* and *M. psychrotolerans* (Fulton, 1943; Emborg et al., 2006). Currently, *M. morganii* is composed of two subspecies: *M. morganii* subsp. *morganii* and *M. morganii* subsp. *sibonii* (Jensen et al., 1992).

Although *M. morganii* typically exists in a commensal relationship within the intestinal tracts of reptiles, mammals, and humans as normal flora, it is considered a rare human pathogen (Bandy, 2020; Zaric et al., 2021). This bacterium can also cause disease in animals (Wei et al., 2023; Zhai et al., 2024). *M. morganii* has been implicated in a wide range of infections, including urinary tract infections (UTIs), nosocomial surgical wound infections, peritonitis, central nervous system infection, endophthalmitis, pneumonia, and chorioamnionitis (Liu et al., 2016; Bandy, 2020). Infections caused by *M. morganii* often have high mortality rates due to the lack of appropriate empirical antibiotic treatment (Erlanger et al., 2019). Although bacteremia caused by *Morganella* spp. is rare, there has been an increase in bloodstream infections caused by *M. morganii* in recent years in Queensland (Laupland et al., 2022). Shockingly, one-fifth of patients infected with *M. morganii* will not survive more than 30 days, underscoring the importance of monitoring this relatively infrequent human pathogen (Laupland et al., 2022).

Carbapenemases play a significant role in multidrug resistance in Enterobacterales, making bacteria resistant to carbapenems and most other β-lactam antibiotics (Sattler et al., 2024). *M. morganii* possesses chromosomally encoded β-lactamases belonging to the AmpC β-lactamase (blaAmpC) family, leading to intrinsic resistance to penicillin, ampicillin, amoxicillin, and most first- and second- generation cephalosporins (Kohlmann et al., 2018; Xiang et al., 2021). Penicillinase is a specific type of β-lactamase that targets penicillins by hydrolyzing the β-lactam ring. A recently study conducted in Germany revealed that a wide diversity of carbapenemases present in *Morganella* spp., such as NDM-1, NDM-5, VIM-1, OXA-48, OXA-181, and OXA-641 (a variant of OXA-372 reported only once in *Citrobacter freundii*) (Bonnin et al., 2024). Conversely, in the Czech Republic, KPC-2 carbapenemase was found to be highly prevalent (Bonnin et al., 2024).

*M. morganii* poses a new clinical treatment challenge due to the continuous acquisition of antimicrobial resistance (AMR) genes, potentially leading to more extensive and challenging multidrug resistance issues (Xiang et al., 2021; Shi et al., 2022). Genetic elements, such as prophages, plasmids, transposons, inserted sequences, and integrons commonly contribute to acquired resistance (Toleman and Walsh, 2011; Shi et al., 2012). Acquired antibiotic resistance in *M. morganii* is primarily mediated by conjugative plasmids, gene mutations and integrons (Liu et al., 2016), which can spread between homogeneous and even heterogeneous bacteria (Liu et al., 2016).

*M. morganii* clinical isolates, harboring various resistance genes to multiple antibiotics, are increasingly reported (Alsaadi et al., 2024). However, large-scale and long-term genomic investigations of *M. morganii* clinical isolates in China are scarce. The aim of this study was to investigate the clinical resistance phenotypes, elucidate the genomic characteristics, and decipher antibiotic resistance mechanisms in *Morganella* spp. sampled between 2016 and 2023.

## Materials and methods

### Study design and bacterial isolates

This was a large-scale retrospective epidemiological study, in which *Morganella* clinical isolates from various clinical samples were included. A total of 218 clinical isolates were obtained from a tertiary teaching hospital affiliated with Capital Medical University between January 1, 2016, and December 31, 2023. Patients’ metadata, including demographic information and dates, were retrospectively gathered from electronic medical records. The bacterial species of all *Morganella* clinical isolates were identified using matrix-assisted laser desorption/ionization-time of flight MS (MALDI-TOF MS) by bioMérieux, France, with *Escherichia coli* ATCC 8739 used as the quality control strain. The isolates were cultured on China blue agar plates (Thermo, USA) at 35 °C for 24 hours.

### Antimicrobial susceptibility testing

Seventeen antimicrobial agents or combinations were selected for drug susceptibility tests based on Clinical and Laboratory Standards Institute (CLSI) guidelines. The susceptibility tests for cefoperazone/sulbactam (SCF), imipenem (IPM), and levofloxacin (LEV) were conducted using the Kirby–Bauer (K-B) disk diffusion method (Oxoid). The minimum inhibitory concentrations (MICs) of the other fourteen drugs, including ampicillin/sulbactam (SAM), amikacin (AK), aztreonam (ATM), ciprofloxacin (CIP), cefotetan (CTT), ceftriaxone (CRO), cefepime (FEP), gentamicin (CN), meropenem (MEM), piperacillin (PRL), trimethoprim sulfamethoxazole (SXT), ceftazidime (CAZ), tobramycin (TOB), and piperacillin-tazobactam (TZP), were determined using the VITEK^®^ 2 AST-GN67 systems (bioMérieux, France).

### Whole-genome sequencing

Genomic DNA was extracted using the Wizard^®^ Genomic DNA Purification Kit (Promega, USA), and the quality was assessed using Qubit 2.0. The DNA was then randomly fragmented using an ultrasonic crusher (Covaris, USA). These processed DNA fragments were utilized for library construction with the NEBNext^®^Ultra™ DNA Library Prep Kit (NEB, USA) for Illumina NovaSeq PE150 sequencing. The raw data obtained was examined and filtered using readfq v10 (https://github.com/lh3/readfq) to eliminate sequences with low-quality (Q ≤ 20) and adapters, as well as remove duplicated reads. The clean data was *de novo* assembled to generate draft genomes using SOAP denovo v2.04 (Li et al., 2010). The gapclose v1.12 tool was employed to refine the initial assembly and fill in any gaps (Xu et al., 2020). For complete genome sequencing, a combination of the Pacific Biosciences sequel platform (PacBio) and Illumina short-read sequencing platform was used, following the method described previously (Zhu et al., 2022).

### Genomic characteristics

The whole-genome average nucleotide identity (ANI), defined as the mean nucleotide identity of orthologous gene pairs shared between two microbial genomes, was calculated using FastANI v1.34 (Jain et al., 2018). Bacterial genomes were annotated using Bakta pipeline v1.9.3 with default parameters (Schwengers et al., 2021). The AMR genes were screened using abricate v1.0.0 (https://github.com/tseemann/abricate) against the NCBI AMRFinderPlus database with default settings (≥80% identity and ≥80% coverage) (Feldgarden et al., 2019). The classification and drug class of obtained AMR genes were further confirmed in the Comprehensive Antibiotic Resistance Database (CARD) (Alcock et al., 2020). Plasmid sequences and types were detected using PlasmidFinder within abricate v1.0.0. Animo acid sequences of gene clusters were extracted and globally aligned with each other, which were then used to generate comparison figures using clinker v0.0.28 (Gilchrist and Chooi, 2021).

### Phylogeny analyses

Single nucleotide polymorphisms (SNPs) in whole genomes were identified using *M. morganii* strain G980 as a reference (GCA_018475185.1). This analysis was performed using Snippy v4.6.0 (https://github.com/tseemann/snippy) as previously reported (Zhou et al., 2021). Loci with high densities of base substitutions in the alignment file, typically indicative of repetitive regions, mobile genetic elements and recombination regions, were identified and subsequently removed through an iterative process using Gubbins v3.3.5 (Croucher et al., 2015). The resulting core SNP alignment was utilized to construct a phylogenetic tree with IQ-TREE v2.0.6 (Nguyen et al., 2015), and the tree was visualized using the Interactive Tree of Life (iTOL) web server (Letunic and Bork, 2021). A SNP matrix based on core-genome multilocus sequence typing (cgMLST) distances was generated using chewBBACA (Silva et al., 2018), with allele calling performed at a 95% loci presence threshold. Genetic clusters were defined as isolates differing by ≤ 10 core-genome single-nucleotide polymorphisms (cgSNPs), while singleton isolates were strains that did not formed any clusters, as determined by ReporTree v2.4.1 (Mixão et al., 2023). A minimum spanning tree was constructed and visualized in GrapeTree v1.5.0 based on pairwise comparison of cgMLST (Zhou et al., 2018). The GFF3 formats of all genomes from this study were annotated using Prokka and analyzed for core-pan genome analysis using the Roary pipeline v3.12.0 (Page et al., 2015).

### Statistical analyses

The continuous variables were summarized as medians with interquartile ranges (IQRs), while the categorical variables were calculated as the percentages of patients in the corresponding category. The heatmap of binary variables was plotted using R package v4.3.3 and clustered based on Euclidean distance. A *P* value of less than 0.05 was considered indicative of statistical significance.

### Ethical approvel

This study was approved by the Ethics Committee of Beijing Chaoyang Hospital, Capital Medical University (2024-ke-381).

## Results

### Epidemiological features of clinical isolates

**Figure 1:**
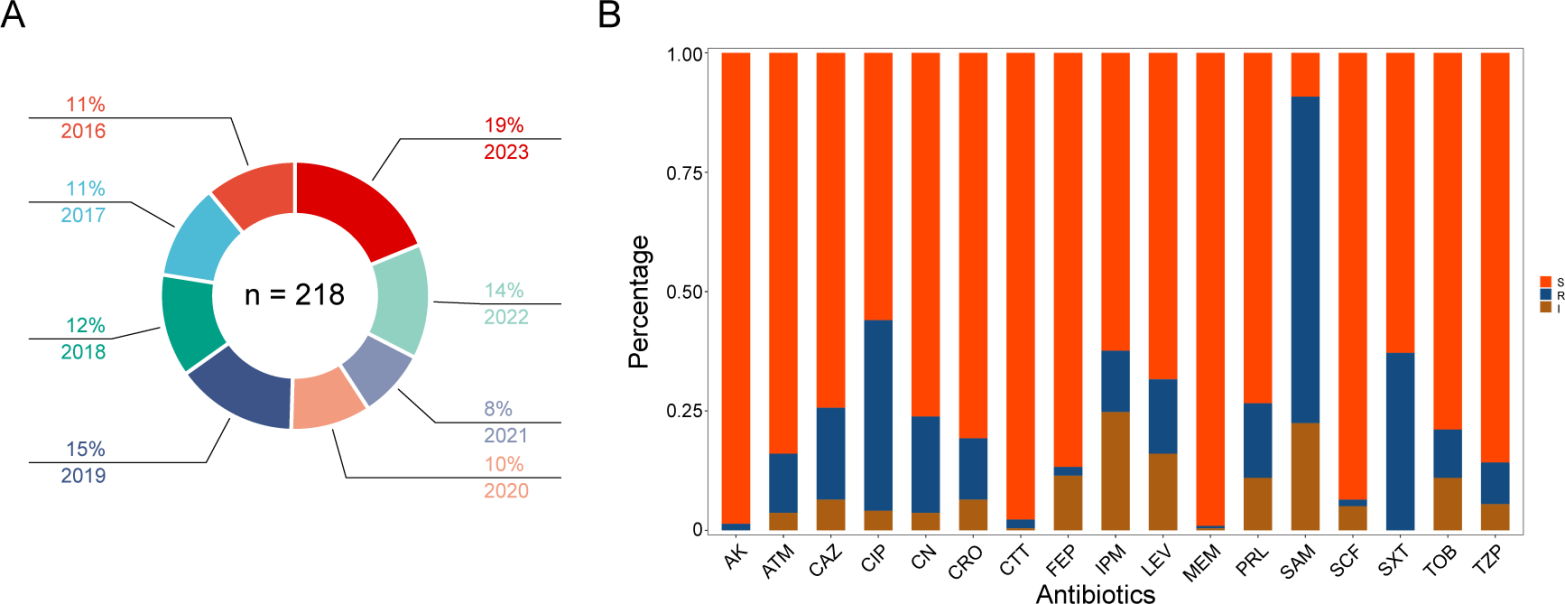
Isolates distribution and antibiotic susceptibility testing. **(A)** Distribution of all *Morganella* clinical isolates across the years. The proportion and year are labeled according to the corresponding color. **(B)** Column chart showing the distribution of susceptibility results from different antibiotics. The ordinate represents the proportion of different phenotypes. S, susceptible; I, intermediate; R, resistance. AK amikacin; ATM, aztreonam; CAZ, ceftazidime; CIP; ciprofloxacin; CN; gentamicin; CRO, ceftriaxone; CTT; cefotetan; FEP, cefepime; IPM, imipenem; LEV, levofloxacin; MEM, meropenem; PRL, piperacillin; SAM, ampicillin/sulbactam; SCF, cefoperazone/sulbactam; SXT, trimethoprim sulfamethoxazole; TOB, tobramycin; TZP, piperacillin-tazobactam.

During the years 2016-2023, a total of 218 *Morganella* strains were isolated from patients (**Figure 1A**). The median age of these patients was 60 year (IQR 54-80), with 62.8% of them being male. The proportion of male patients were significantly higher than that of female patients (*P* = 0.0001). *Morganella* spp. were predominantly isolated from urine (48.2%), followed by respiratory tract samples (sputum and bronchoalveolar lavage fluid) (16.5%), semen (6.0%), blood (5.5%), wound secretion (5.0%), and bile (3.2%). These patients were primarily admitted to the departments of urology (37.6%), neurosurgery (7.8%), emergency (5.0%), and surgical intensive care unit (5.0%). The *Morganella* spp. were often co-infected with *Escherichia coli* (13.7%), followed by *Pseudomonas aeruginosa* (11.5%), *Klebsiella pneumoniae* (6.9%), and *Proteus mirabilis* (4.1%).

### Antimicrobial susceptibility profiles

**Figure 2:**
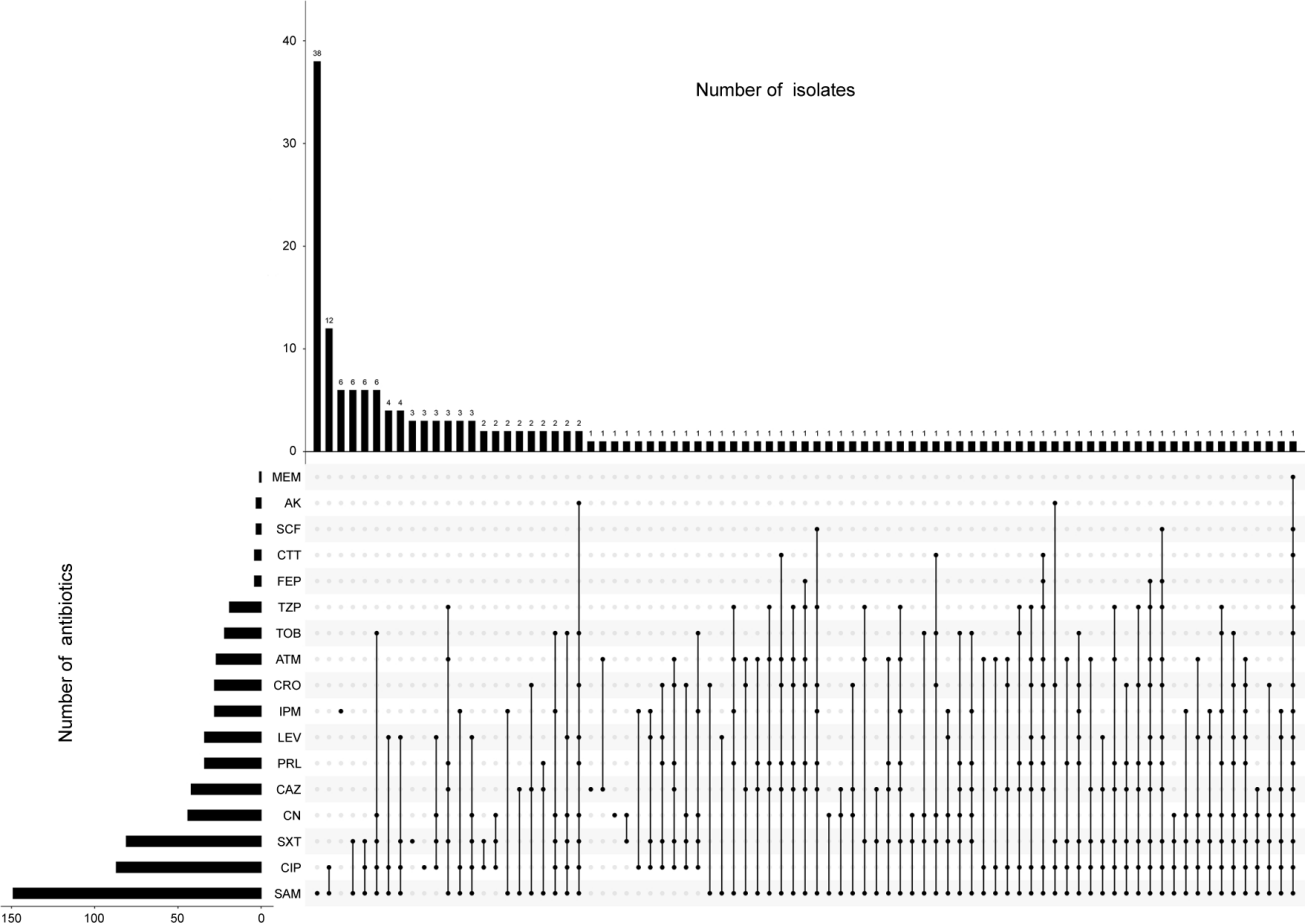
UpSetR plot indicating the counts and correlation of antibiotic resistance among *Morganella* clinical isolates. Only susceptibility testing results that have been determined to be resistant according to Clinical and Laboratory Standards Institute guidelines are included.

Results from the antimicrobial susceptibility testing of *Morganella* strains (**Supplmenetary Table S1**) revealed varying proportions of each antimicrobial resistant phenotype. The most common resistant phenotypes were SAM (68.3%), CIP (39.9%), SXT (37.2%), CN (20.2%), CAZ (19.3%), LEV (15.6%), PRL (15.6%), CRO (12.8%), IPM (12.8%), ATM (12.4%), TOB (10.1%), TZP (8.7%), CTT (1.8%), FEP (1.8%), AK (1.4%), SCF (1.4%), and MEM (0.5%) (**Figures 1B**, **2**). The most prevalent resistant profile was resistant only to SAM (n=38), followed by resistant to both SAM and CIP (n=12) (**Figure 2**). There were four distinct resistance profiles [(IPM), (SAM and SXT), (SAM, CIP and SXT), (SAM, CIP, SXT, CN, and TOB)], with six strains exhibiting each phenotype. Additionally, sixty-two strains showed unique resistance profile, each with its own set of resistant phenotypes (**Figure 2**). These results indicated that *Morganella* isolates exhibit a diverse range of drug resistance profiles. When resistant rates of each antimicrobial agent were analyzed by year, it was found that *Morganella* strains in 2018 had the lowest resistant rates (**Supplementary Figure S1**), while the highest resistant rate was observed in isolates from 2020. Despite fluctuations in resistance rates during the COVID-19 pandemic, the overall trend indicated an increase in drug resistance rate.

### Genome sequencing and comparison

According to the type of antimicrobial resistance profile, a total of 105 isolates were selected for whole genome sequencing, which included five complete and 100 draft genomes. Among the five complete genomes, two strains (CY46256 and CY54639) contained a single chromosome and two circular plasmids, while two strains (CY69118 and CY74555) contained a single chromosome and one circular plasmid. The genome sizes of the 100 draft genomes ranged from 3.66 to 4.73 Mb, with N50 values ranging from 87,370 to 2,678,398 bp. The number of contigs per genome ranged from 5 to 92, with median number of 33. These indicators demonstrated that the quality of the genomes obtained in this study was superior than those found in the NCBI public database. The G+C content of each genome ranged from 50.3 to 51.3 mol%. Results from genomic prediction and annotation revealed that the number of genes in the genomes ranged from 3,512 to 4,264.

**Figure 3:**
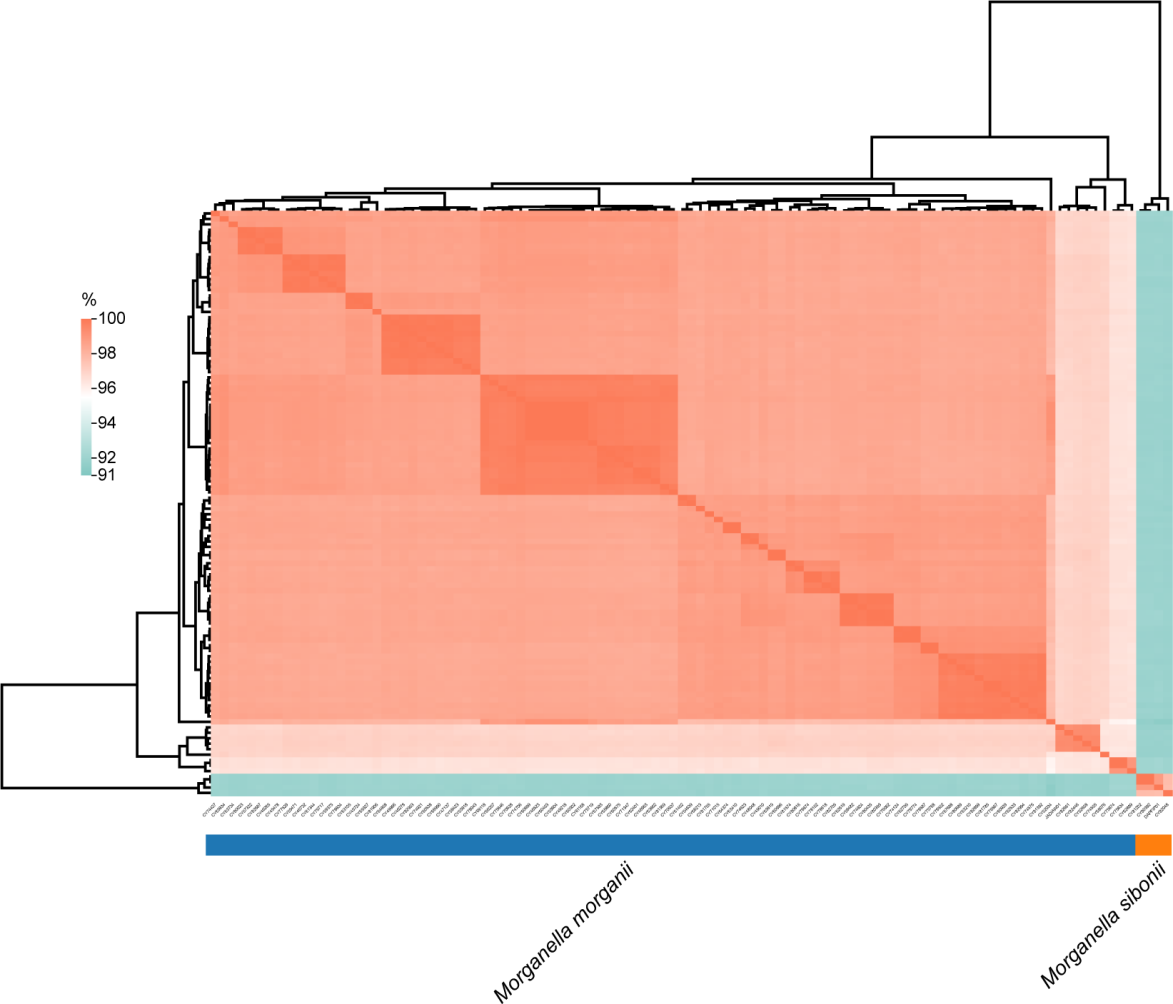
Analysis of average nucleotide identity among *Morganella* clinical isolates. The values obtained were clustered based on Euclidean distance. The average nucleotide identities ranged from 91% to 100%.

To further investigate the genomic distance, a similarity matrix based on ANI was estimated with strain *M. morganii* subsp. *morganii* G980 (GenBank accession number JADIAW1) and strain *M. morganii* subsp. *sibonii* 8481 (GenBank accession number DAPFIP01) as the references. Three isolates of *M. morganii* (CY81202, CY80392, and CY82048) collected in 2023 from this study were clustered and shared 97.7-99.9% identity with strain 8481, suggested they belong to the *M. morganii* subsp. *sibonii* group (**Figure 3**). The other 102 isolates from our study formed another cluster together with strain G980, sharing 95.7 to 99.9% identity each other, indicating they all belonged to the *M. morganii* subsp. *morganii* group. However, the ANI results between members of *M. morganii* subsp. *sibonii* and *M. morganii* subsp. *morganii* groups showed only 91.5 to 92.2% identity, indicating that these two subspecies could be considered as two different species.

### Phylogenomic analysis

**Figure 4:**
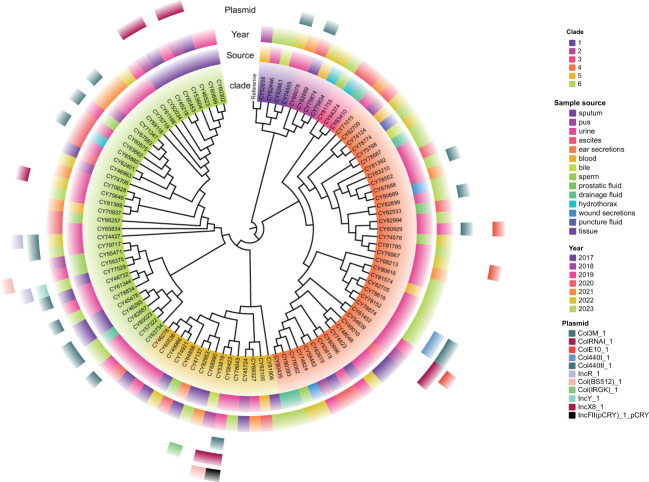
Maximum likelihood phylogenetic tree of 102 *M. morganii* clinical strains based on the core genome. *Morganella morganii* subsp. *morganii* strain G980 (JADIAW010000001.1) was used as the reference to root the tree. The colored rings, from the inside out, represent the clades, sample source, collected year, and distribution of plasmid types.

A total of 2536 alleles were called using the chewBBACA cgMLST schema for these 102 isolates of *M. morganii*, based on 95% loci presence (**Supplementary Figure S2**). The constructed core-genome phylogenetic tree indicated that these isolates could be divided into six clades (**Figure 4**), with the majority of them belonging to clade 4 (37.3%, 38/102) and clade 6 (37.3%, 38/102). The phylogenetic tree also revealed that multiple evolutionary clades were not congruent with isolated years and sample types, and independent of plasmid types.

**Figure 5:**
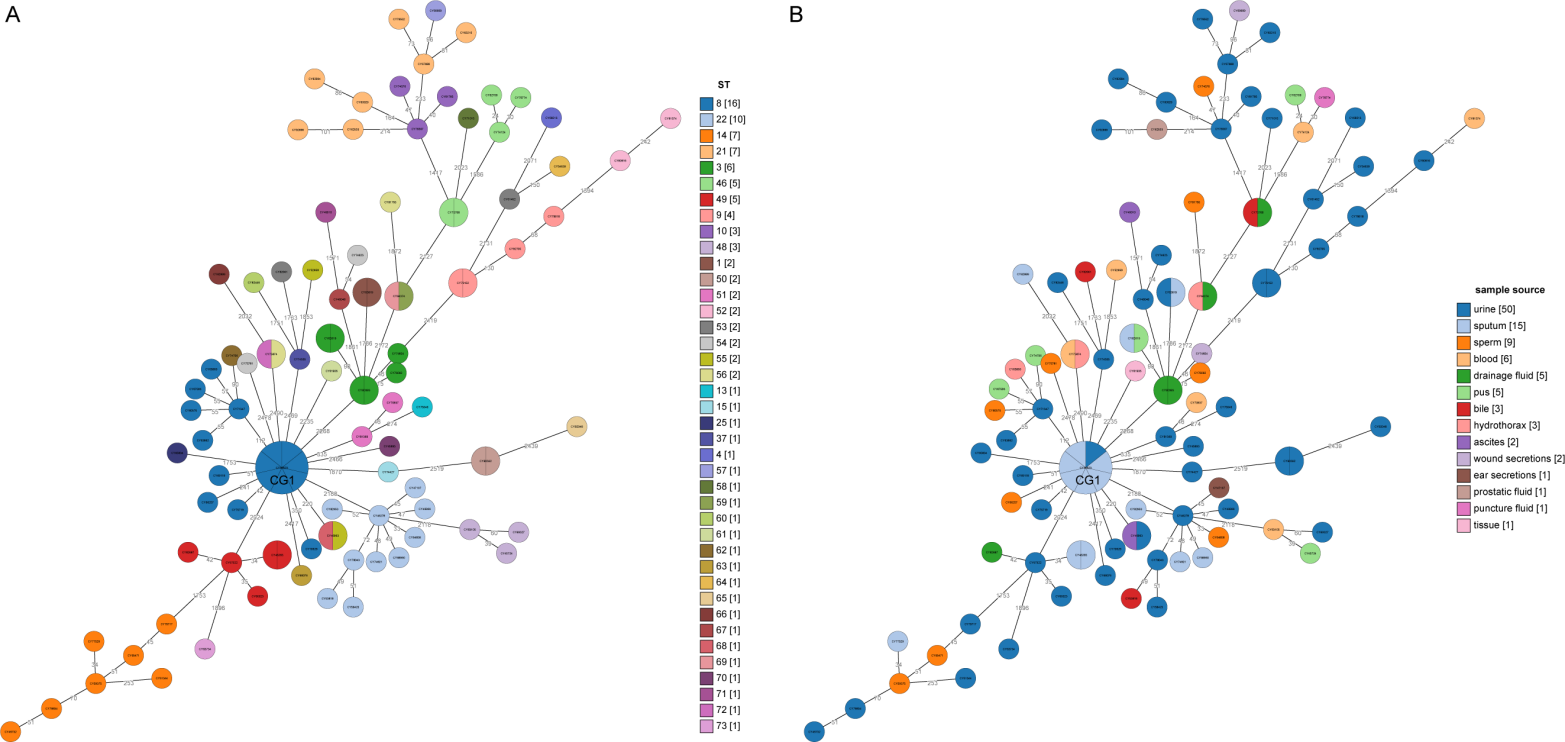
Minimum spanning trees based on core genome MLST analysis. **(A)** The circles on the trees are color-coded to represent ST determined by ≤10 SNPs. The numbers in square brackets represent the number of strains in each cluster singleton. **(B)** The circles are color-coded based on the sample source from which the strains were collected, with the corresponding number of strains from each year shown in square brackets. The number of SNP differences between adjacent strains is labeled on the lines connecting them.

A minimum spanning tree (MST) was constructed based on these cgSNPs. The number of pairwise SNPs of isolates from this study ranged from 1 to 2519, with a median of 79. These isolates were initially clustered based on a 100 cgSNP cutoff, resulting in 21 clusters and 26 singletons (**Figure 4**), indicating genome heterogeneity. Then, based on a threshold of ≤ 10 cgSNPs, 11 genetic clusters (GCs 1-11) and 78 singletons were observed (**Figure 5**). Except for genetic cluster 1 (GC1) including seven isolates, the other ten genetic clusters (GC2-GC11) contained only two isolates. Additionally, the correlation between genetic clusters and ST was investigated, suggesting strains of CGs are not exactly grouped together by ST (**Figure 5A**). However, seven isolates of GC1 all belonged to ST. For the other genetic clusters, seven of them belonged to the same ST. Strains of CGs are also not exactly grouped together by sample source (**Figure 5B**). We further investigated whether there were nosocomial infections, which revealed that isolates of seven genetic clusters out of these eleven genetic clusters had strains from the same patient. For the other four genetic clusters, stains of three GCs (GC4-6) were isolated from two patients three months apart, and strains of GC11 were isolated from two patients three years apart.

### Core and pan genome analyses

As the number of genomes increased, the number of core genes stabilized, while the number of pan genes continued to increase. A total of 13202 gene clusters were identified in these 102 *M. morganii* clinical isolates (**Supplementary Figure S3**). Out of these, 2549 genes were defined as core genes, indicating their presence in 102-105 isolates (**Supplementary Figure S4**). There were 357 genes shared by 96-100 isolates, defined as soft-core genes (> 95% isolate prevalence). Additionally, the majority (68.0%, 8975/13202) of the genes were defined as cloud genes, which were presented in fewer than 15 isolates (**Supplementary Figure S4**).

Furthermore, we compared the core and pan genomes of different clades of *M. morganii*. A total 3,102 genes were shared by these six clades (**Supplementary Figure S5A**). The number of unique genes of clades 1-6 were 2514, 799, 2089, 833, 436, and 86, respectively. Among the unique genes of clades 1-6, 27.1%, 16.8%, 11.6%, 17.6%, 13.8%, and 12.7%, respectively, were not assigned to any COG by EggNOG (**Supplementary Figure S5A**). Of these assigned genes, most belonged to S (unknown function) COG, followed by L (replication, recombination, and repair), K (transcription), and M (cell wall/membrane/envelope biogenesis) COGs (**Supplementary Figure S5B**).

### Antimicrobial resistance genes

**Figure 6:**
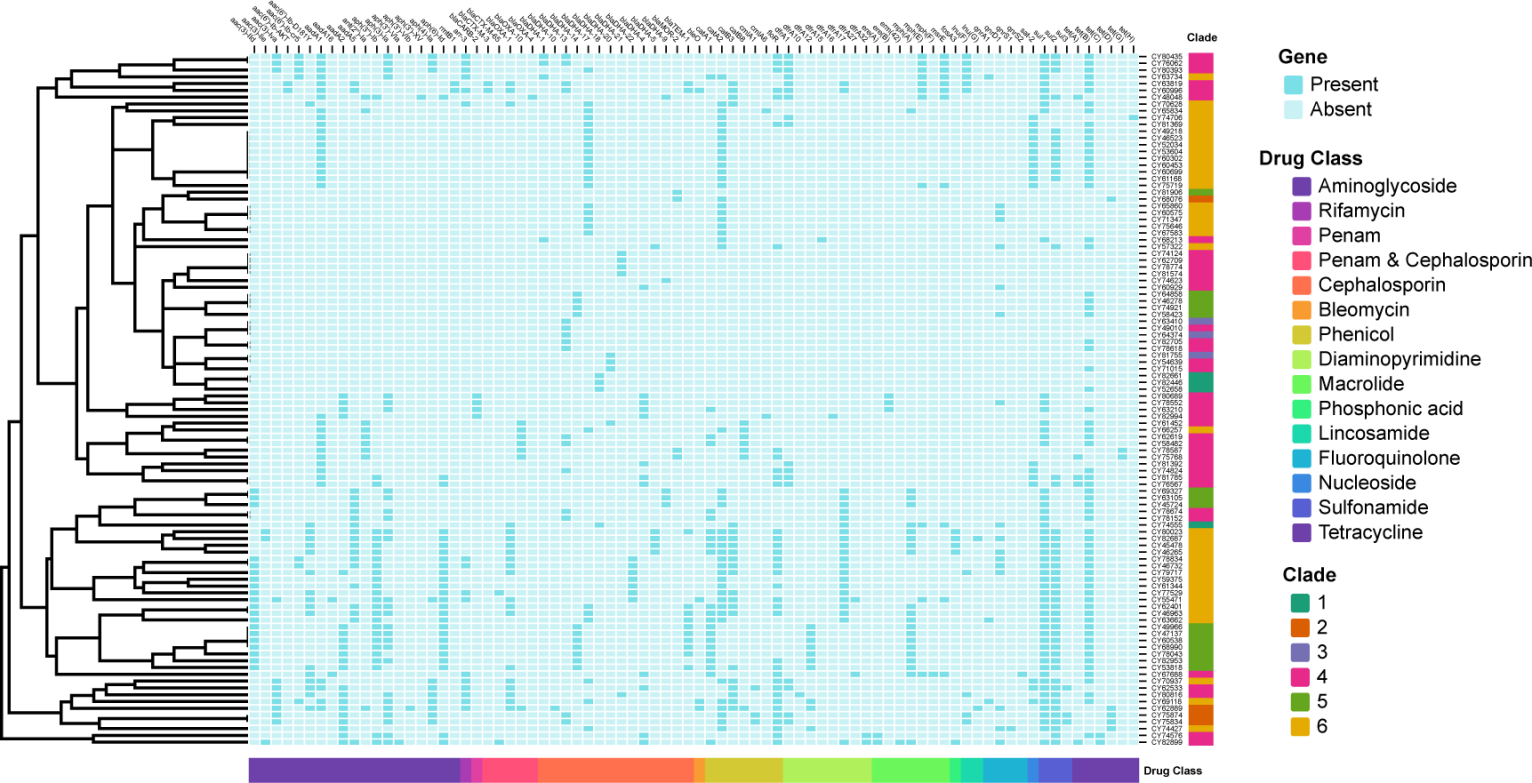
Binary heatmap displaying the presence and absence of antimicrobial resistance (AMR) genes. The AMR genes are categorized into fifteen drug classes labeled on the *x*-axis. The clades are labeled on the *y*-axis. Baby blue squares indicate the absence of the corresponding AMR genes, while dark blue squares indicate their presence.

To provide a genomic context for AMR genes, genomes were screened against the NCBI AMRFinderPlus database using a relatively strict cutoff (90% identity and 80% coverage). Putative AMR genes were identified in all 102 sequenced isolates (*M. morganii*), with number of AMR genes ranging from 1 to 23 (median of 8) for each isolate. The most prevalence AMR gene was the *tet(B)* (65.7%, 67/102), followed by *sul1* (52.9%, 54/102), *catA2* (45.1%, 46/102), *sul2* (45.1%, 46/102), *floR* (35.3%, 36/102), and *aadA1* (33.3%, 34/102). A total of 80 AMR-related genes were detected in these 102 *M. morganii* clinical isolates (**Figure 6**), with 26 (32.5%, 26/80) of these AMR genes had a prevalence of more than 10%.

Based on the AMR mechanism, the 80 detected AMR genes were further categorized into 15 drug-related classes (**Figure 6**). The most prevalent drug resistance class was caused by β-lactam resistance, including penam, penam & cephalosporin, cephalosporin. Among them, the *bla*_DHA/MOR_-like genes were presented in all 102 isolates, with *bla*_DHA-17_ being the most prevalent type (25.5%, 26/102). Extended-spectrum β-lactamases (ESBLs) or cepahlosporinases were found in 48 (47.1%) of these 102 isolates, including *bla*_OXA-1_ (19.6%, 20/102), *bla*_TEM-1_(11.8%, 12/102), *bla*_OXA-10_ (6.9%, 7/102), *bla*_CARB-2_ (3.9%, 4/102), *bla*_CTX-M-3_ (2.0%, 2/102), *bla*_CTX-M-65_ (2.0%, 2/102), and *bla*_OXA-4_ (1.0%, 1/102). Furthermore, a total of 241 aminoglycoside phosphotransferase related genes were detected, with *aadA1* (33.3%, 34/102), *aph(3)-Ia* (27.5%, 28/102), *aph(3’’)-Ib* (25.5%, 26/102), and *aph(6)-Id* (25.5%, 26/102) being the most prevalent. Sixteen isolates (15.7%) contained rifamycin-resistant genes, all caused by presence of *arr-3*. The *bleO* gene (resistant to bleomycin) was found in four isolates. Phenicol-related resistant genes were presented in 76 isolates, mainly including *catA2* (46), *floR* (36), *catB3* (22), and *catA1* (22). Diaminopyrimidine-related resistant genes were presented in 57 isolates, mainly including *dfrA17* (23) and *dfrA1* (17). A total of 39 isolates had macrolide-related resistant genes, including *mph(A)* (21), *mph(E)* (11), and *msrE* (11), while 103 isolates contained sulfonamide-related resistant genes comprised of *sul1* (54), *sul2*(46), and *sul3*(3). For fluoroquinolone resistance, 21 related genes were detected, including the *qnrA1* (3), *qnrD1* (16), *qnrS1*(1), and *qnrS2* (1). For lincosamide resistance, genes *lnu(F)* and *lnu(G)* were identified in ten and two isolates, respectively. Gene *sat-2* was detected in 18 isolates, which resulted in resistance to nucleoside antibiotic. Additionally, a total of 82 isolates contained tetracycline (tet)-related resistant genes, including *tet(B)* (67), *tet(D)* (5), *tet(A)* (5), *tet(C)* (2), *tet(G)* (2), and *tet(H)* (1).

### Plasmid characterization

A total of 41 plasmids were detected in 32 clinical isolates (31.4%, 32/102), with eight isolates having two plasmids and two isolates having three plasmids (**Figure 4**). The most prevalent plasmid types were Col3M (15.7%, 16/102), followed by ColRNAI (7.8%, 8/102), ColE10 (3.9%, 4/102), Col440I (2.0%, 2/102), Col440II (2.0%, 2/102), IncR (2.0%, 2/102), and Col (BS512) (2.0%, 2/102). In addition, five plasmid types, including Col (IRGK), IncFII (pCRY), IncL/M (pOXA-48), IncX8, and IncY, were only detected in one isolate each.

**Figure 7:**
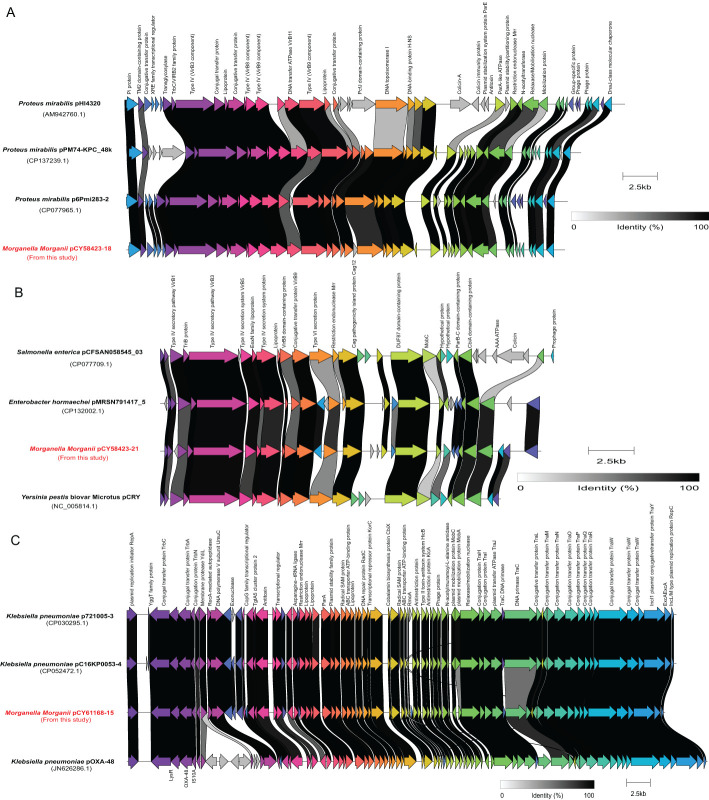
Schematic representations and genetic context comparisons among the plasmids. Coding sequences are depicted by arrows, which also show the direction of transcription. The proteins being encoded are labeled above their respective arrows. Unlabeled arrows indicate genes coding for hypothetical proteins. The length of the arrows is proportional to the gene’s length. The color of the lines connecting genes reflects their similarities. Plasmids identified in this study were labelled with red font.

Three plasmids, each more than 20 kb in length, were compared with their closely related counterparts (**Figure 7**). The genome of strain CY58423 contained three different type plasmids (IncX8, IncFII (pCRY) and Col3M). The IncX8 type plasmid (pCY58423-18, 36,289 bp) was found to be closely related to the *Proteus mirabilis* pHI4320 plasmid (100% coverage and 99.2% identity), followed by *Proteus mirabilis* p6Pmi283-2 and *Proteus mirabilis* pPM74-KPC_48k plasmids. The majority of genes within the backbone structure of this plasmid were associated with conjugal transfer elements, such as type IV and conjugative transfer proteins, and did not contain AMR genes (**Figure 7A**). The IncFII (pCRY) type plasmid (pCY58423-21, 21,742 bp) was closely related to the p*CRY* plasmid of *Yersinia pestis* biovar Microtus str. 91001. It mainly encoded proteins related to the type IV secretion system and conjugative transfer (**Figure 7B**). Strain CY61168 carried an IncL/M type plasmid (pCY61168-15, 61,881 bp), closely related to the pOXA-48 plasmid of strain *Klebsiella pneumoniae* Kp11978, with a 100% identity. This was followed by *Klebsiella pneumoniae* p721005-3 and pC16KP0053-4 plasmids. Aside from conjugative transfer proteins, this plasmid also encoded other functional proteins, such as type II toxin-antitoxin system, lipoprotein, and radical SAM protein (**Figure 7C**). However, these three larger plasmids did not contain genes encoding for carbapenemase, which were also absented in other plasmids or genomes from this study, but could be presented in their closely related plasmids.

## Discussion

The alarming increase in rare opportunistic microbial infections worldwide is a cause for concern. However, the genomic epidemiology of *Morganella* spp. recovered from clinical samples have been rarely explored in China, and worldwide (Xiang et al., 2021; Bonnin et al., 2024). In this study, we collected the drug-resistant phenotype of 218 clinical isolates and conducted a large-scale genomic study on 105 *M. morganii* clinical isolates in Beijing between 2016 and 2023 to provide an overview of epidemiology and genomic characteristics of *M. morganii*. Based on a cutoff at 95% ANI (Richter and Rosselló-Móra, 2009), our research suggested that members of *M. morganii* subsp. *morganii* and *M. morganii* subsp. *sibonii* could be reclassified as two species, which was also supported by phylogenetic reconstructions. Therefore, the new combinations for species are proposed, namely *Morganella morganii* comb. nov and *Morganella sibonii* comb. nov.

The proportion of different types of infections may vary from hospital to hospital. The most common infection caused by *M. morganii* in this study was urinary tract infection, followed by respiratory tract infection, which was basically consistent with previous reports (Xiang et al., 2021). Shunt fluid infection was the most common clinical infection site observed in the First Affiliated Hospital of Sun Yat-sen University, China (Xiang et al., 2021). Bile was the second major source of infection based on data from the General Hospital of Ningxia Medical University, China (Xiang et al., 2021). Additionally, blood and semen infections are increasing and deserve more attention (Laupland et al., 2022).

*M. morganii* is accumulating both intrinsic and acquired multidrug resistance genes, leading to higher morbidity and mortality rates in infections caused by this bacterium (Bandy, 2020). In this study, the antibiotic resistance profiles of the strains showed a high rate (94.5%) of antibiotic resistance, with only 12 strains being fully susceptible to all tested antibiotics. The antibiotic resistance rates of most antibiotics tested were significantly higher compared to other three tertiary hospitals in China (**Supplementary Table S2**) (Xiang et al., 2021). However, the resistance rates of SXT (56.0% V.S. 37.2%), CAZ (34.0% V.S. 19.3%), and CRO (22.0% V.S. 12.8%) of strains collected from General Hospital of Ningxia Medical University were higher than those from this study. The differences in knowledge of antimicrobial clinician, and economic and medical conditions, could explain the varied antibiotic resistance profiles across regions (Zhen et al., 2019). In a recent study, Rémy A Bonnin and colleagues showed that intrinsic resistance to tetracycline was found only in *M*. *sibonii* (Bonnin et al., 2024), while we found tetracycline-related resistance genes in 82 *M. morganii* isolates in our study. The prevalence of carbapenem-resistant Enterobacterales has increased rapidly, and has become a serious threat to public health (Cui et al., 2019). Although a few studies revealed that the carbapenem-resistant *M. morganii* has been detected with a low prevalence (Xiang et al., 2021; Bonnin et al., 2024), the β-lactam resistance genes in this study were comprised of ESBL, cephalosporinase, and penicillinase genes, with the most prevalent carbapenem-resistant genes, such as *bla*_KPC-2_, *bla*_NDM-1_, and *bla*_OXA-48_, not being found. However, *Morganii* spp. was on its way to becoming a next “superbug”, which deserves our constant attention. In addition, none of the isolates from this study contained any virulence genes when compared against the virulence factor database.

Only eleven genetic clusters, which included 27 isolates, were defined based on ≤ 10 SNPs. Seven of these genetic clusters were collected from the same patients, indicating a high genomic diversity among different hosts of *M. morganii* isolates. A subset of *M. morganii* clinical isolates (7 strains) from the same patient were identified with very similar genetic backgrounds (< 10 SNPs accumulated over three years), suggesting the stability of *M. morganii* isolates within the same host. Additionally, a cutoff at 20 SNPs per genome was established to distinguish isolates from an outbreak in *Klebsiella penumoniae* (David et al., 2019), which also applied to *M. morganii* (Bonnin et al., 2024). We identified four genetic clusters, each including two isolates, that were obtained from different patients with time intervals varying from 1 month to 3 years. These data suggested possible nosocomial transmission of the isolates from these four clusters, especially for isolates of two clusters sampled from the same department.

In conclusion, we conducted a detailed analysis of a large-scale genomic study on *Morganella* clinical isolates and recommended a reclassification of the species *M. morganii*. The isolates examined in this study showed high rates of antibiotic resistance, including the emergence of multidrug-resistant strains. The minimal number of cgSNP differences observed indicated possible instances of in-hospital transmission. While antibiotic-resistant *M. morganii* is not currently a significant issue in China, ongoing surveillance is vital to monitor the prevalence and characteristics of multidrug-resistant strains, especially those resistant to carbapenems. Such vigilance will aid in the effective management and prevention of potential outbreaks in the future.

## Data Availability

The genomes of *Morganella* clinical isolates that were sequenced in this study have been deposited in the NCBI SRA repository under the accession number PRJNA1119085.
