# Supplementary material for: Genomic epidemiology and antimicrobial resistance of Morganella clinical isolates between 2016 and 2023
Source: Front Cell Infect Microbiol. 2025 Jan 31;14:1464736. doi: 10.3389/fcimb.2024.1464736 (PMC11826060; doi:10.3389/fcimb.2024.1464736)
Supplement: Supplementary file 1 [file DataSheet1.docx]

**Supplementary materials**


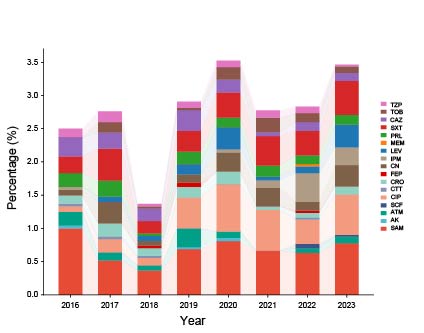


Figure S1 Antibiotic resistance distribution per year. The *y*-axis represents the sum of drug resistance rates that is equal to the sum of the resistance rates of each antibiotic. AK amikacin; ATM: aztreonam; CAZ: ceftazidime; CIP; ciprofloxacin; CN; gentamicin; CRO: ceftriaxone; CTT; cefotetan; FEP: cefepime; IPM: imipenem; LEV: levofloxacin; MEM: meropenem; PRL: piperacillin; SAM: ampicillin/sulbactam; SCF: cefoperazone/sulbactam; SXT: trimethoprim sulfamethoxazole; TOB: tobramycin; TZP: piperacillin-tazobactam.


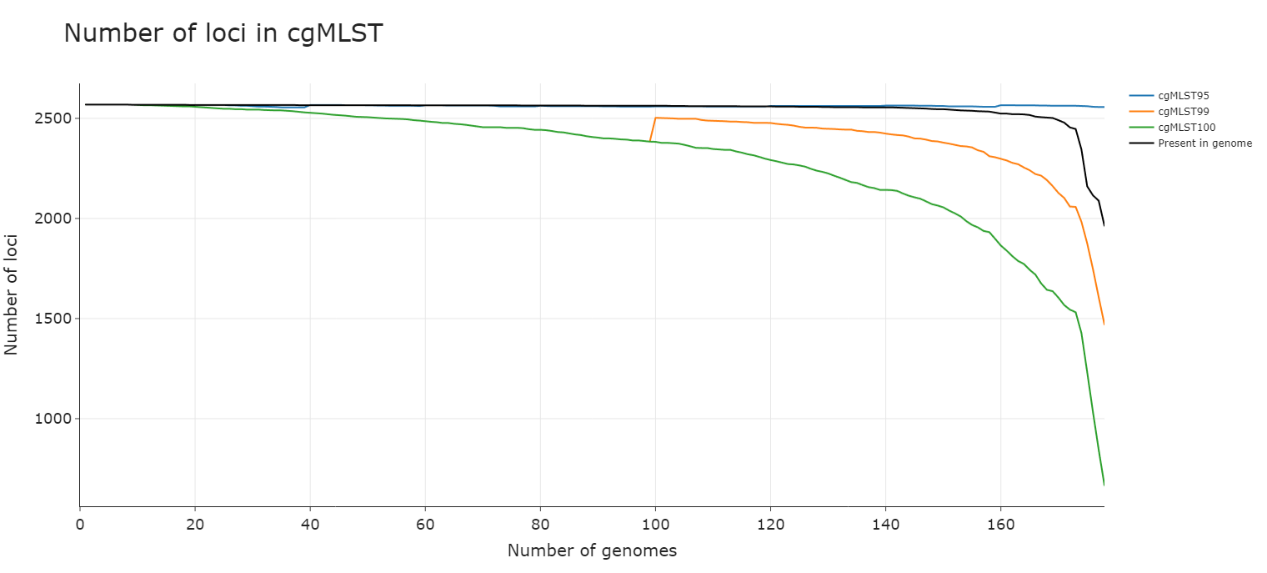


Figure S2 Comparison of the loci presence thresholds. The set of loci in the core genome is determined by running the ExtractCgMLST module.


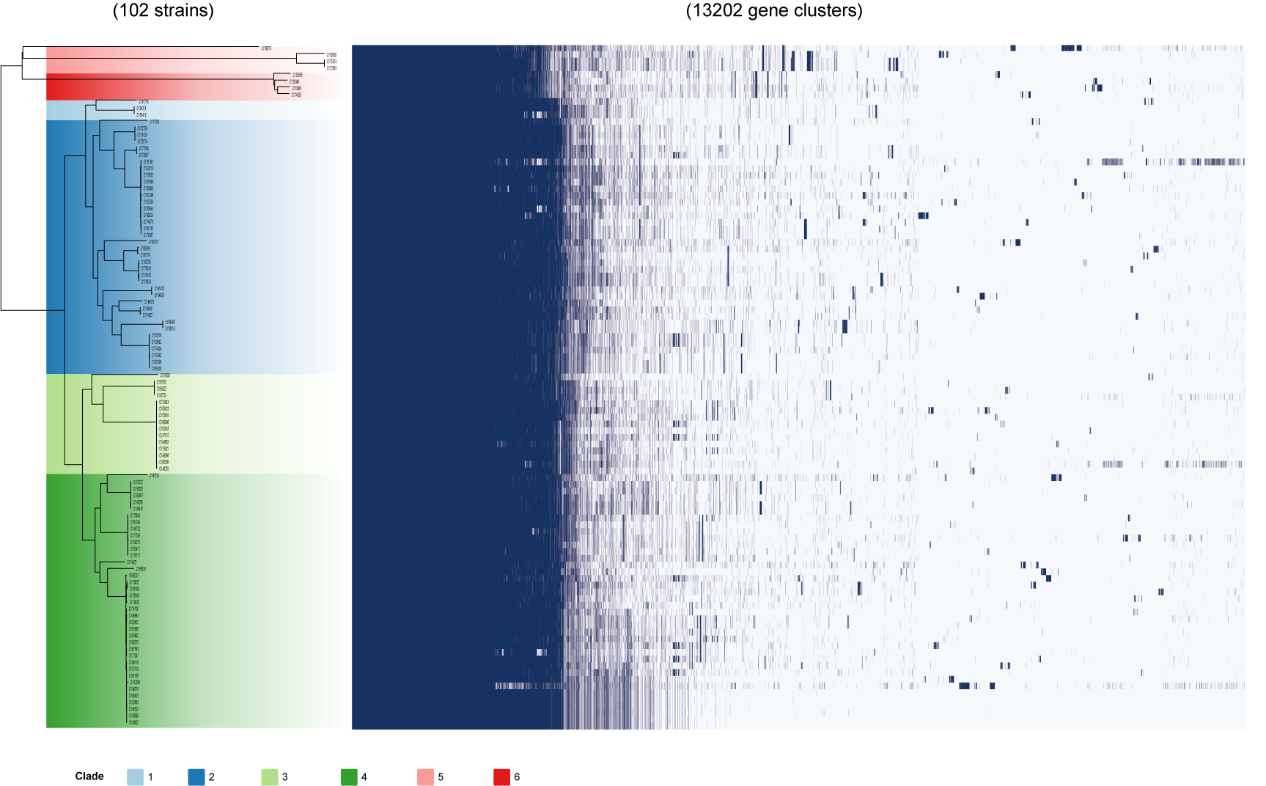


Figure S3 Pangenome analysis of genomes of *M. morganii* clinical strains. The phylogenomic tree was constructed based on the core orthologous groups. The clades were labeled with corresponding colors. The blue squares represent that the corresponding genes are absent. The white squares represent that the corresponding genes are present.


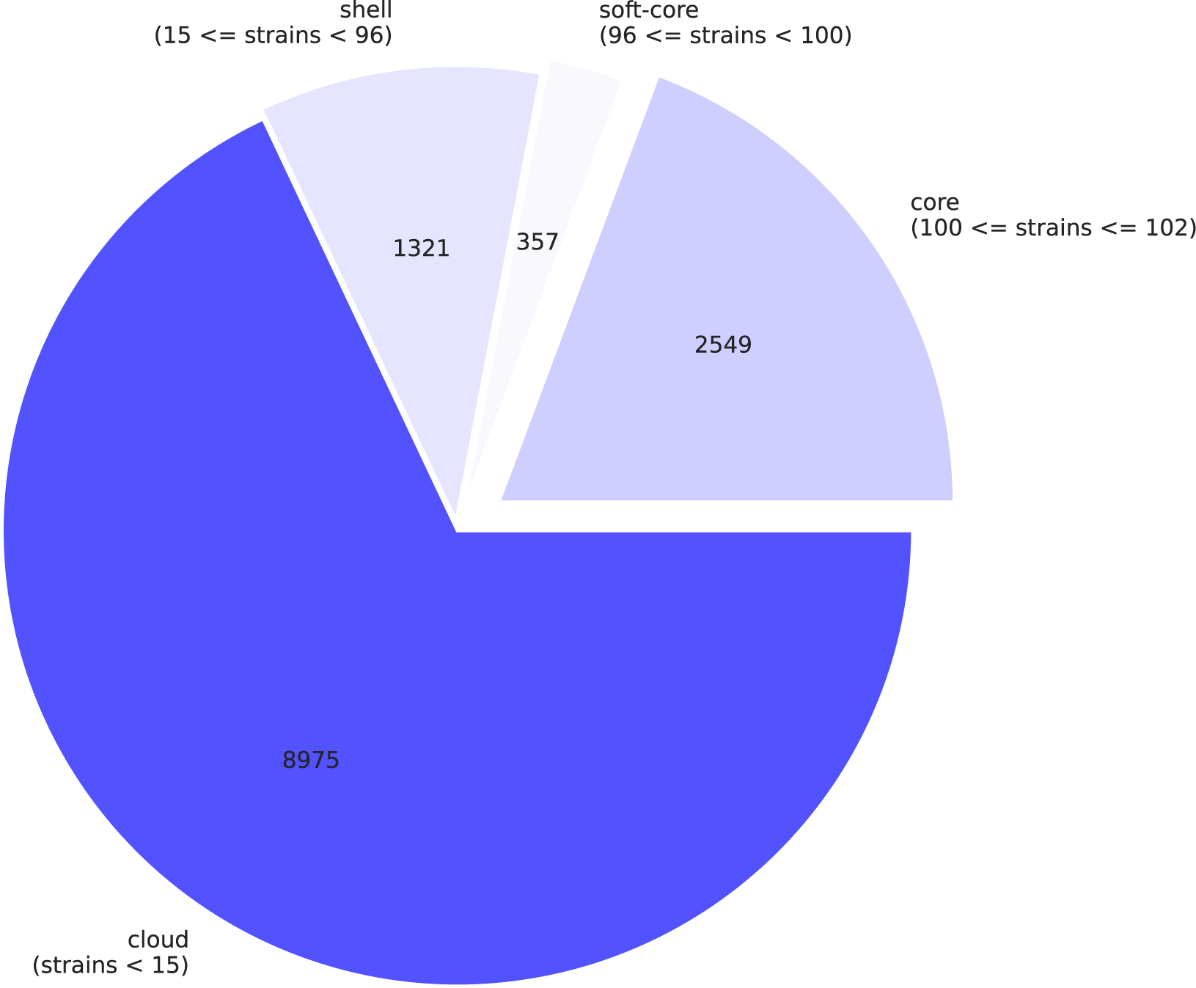


Figure S4 Number of core and accessory genes of *M. morganii* clinical strains.


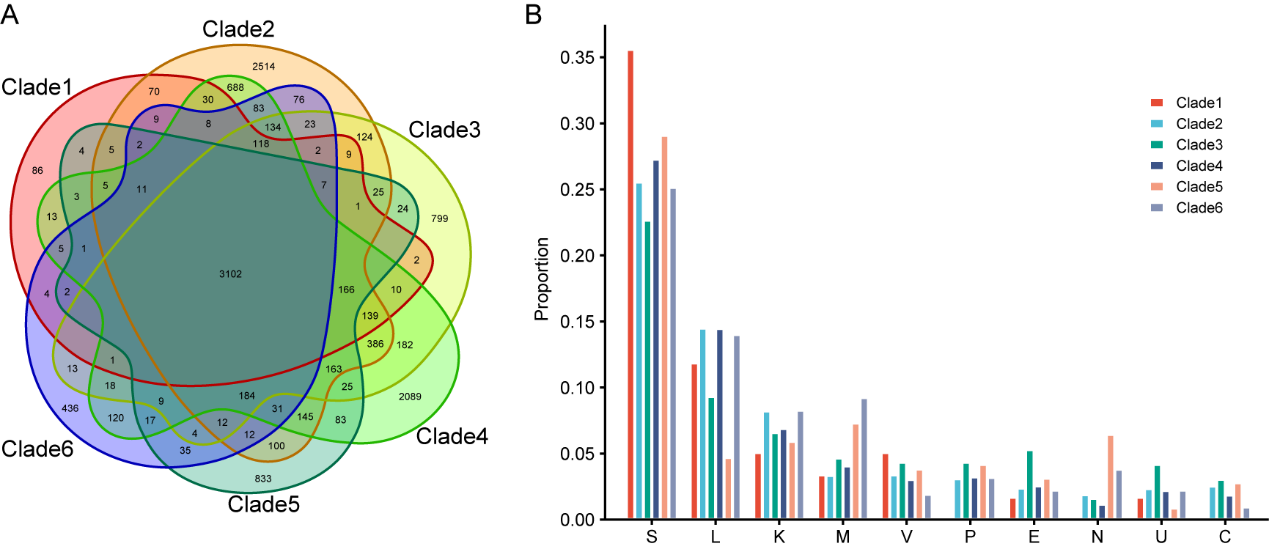


Figure S5 Pan-genomic comparisons among the six clades. (A) Venn diagram illustrating the numbers of core and unique homologous clusters from different clades. (B) Comparisons of the top ten COG functional classifications of core-specific clusters within each clade. S: Function unknown; L: Replication, recombination, and repair; K: Transcription; M: Cell wall/membrane/envelope biogenesis; V: Defense mechanisms; P: Inorganic ion transport and metabolism; E: Amino acid transport and metabolism; N: Cell motility; U: Intracellular trafficking, secretion, and vesicular transport; C: Energy production and conversion.

Table S1 Antibiotic resistance results of the 218 *M. morganii* isolates involved in this study

| **Isolates** | **SAM** | **AK** | **ATM** | **SCF** | **CIP** | **CTT** | **CRO** | **FEP** | **CN** | **IPM** | **LEV** | **MEM** | **PRL** | **SXT** | **CAZ** | **TOB** | **TZP** |
| --- | --- | --- | --- | --- | --- | --- | --- | --- | --- | --- | --- | --- | --- | --- | --- | --- | --- |
| CY39272 | R | S | S | S | S | S | S | S | S | S | S | S | S | S | S | S | S |
| CY39325 | R | S | R | S | I | S | I | S | S | I | S | S | R | R | R | S | R |
| CY39655 | R | S | S | S | S | S | S | S | S | S | S | S | S | S | S | S | S |
| CY39819 | R | S | R | S | S | S | S | S | S | R | S | S | R | S | I | S | R |
| CY40378 | R | S | S | S | S | S | S | S | S | S | S | S | S | S | S | S | S |
| CY40609 | R | S | S | S | S | S | S | S | S | S | S | S | S | R | S | S | S |
| CY40873 | R | S | R | S | I | S | I | S | S | S | S | S | R | R | R | S | R |
| CY41128 | R | R | S | S | R | S | R | S | S | S | I | S | S | R | S | S | S |
| CY42015 | R | S | S | S | S | S | S | S | S | S | S | S | S | S | I | S | S |
| CY42918 | R | S | S | S | S | S | S | S | S | S | S | S | S | R | S | S | S |
| CY42966 | R | S | S | S | S | S | S | S | S | S | S | S | S | S | S | S | S |
| CY43386 | R | S | R | I | S | R | R | S | S | S | S | S | R | S | R | S | I |
| CY43446 | R | S | S | S | S | S | R | S | S | S | S | S | I | S | R | S | S |
| CY43479 | R | S | S | S | S | S | I | S | S | S | S | S | R | S | R | S | S |
| CY43558 | R | S | S | I | S | S | S | S | S | S | S | S | S | S | S | S | S |
| CY43559 | R | S | S | S | S | S | S | S | S | S | S | S | S | S | S | S | S |
| CY43625 | R | S | R | S | R | S | S | S | S | S | S | S | S | S | R | S | S |
| CY43709 | R | S | S | S | S | S | S | S | S | S | S | S | S | S | S | S | S |
| CY44082 | R | S | S | S | S | S | S | S | S | S | S | S | S | S | S | S | S |
| CY44188 | R | S | S | S | I | S | S | S | S | S | I | S | S | R | S | S | S |
| CY44193 | R | S | S | S | S | S | S | S | S | S | S | S | I | S | I | S | I |
| CY44470 | R | S | S | S | S | S | S | S | R | S | S | S | I | S | R | S | S |
| CY44471 | R | S | S | I | S | S | S | S | R | S | S | S | I | S | S | S | S |
| CY44496 | R | S | S | S | S | S | S | S | S | I | S | S | S | S | S | S | S |
| CY45301 | R | S | S | S | S | S | S | S | S | S | S | S | S | S | S | S | S |
| CY45478 | R | S | S | S | R | S | R | S | S | S | S | S | R | R | R | I | S |
| CY45724 | R | S | I | S | S | S | R | I | R | S | S | S | I | S | R | I | I |
| CY45893 | I | S | S | S | R | S | S | S | R | S | R | S | S | R | S | I | S |
| CY46265 | R | S | I | S | R | S | R | I | S | S | S | S | R | R | R | I | R |
| CY46278 | R | S | R | S | I | S | I | I | S | S | S | S | R | S | R | S | R |
| CY46523 | R | S | R | S | I | S | S | I | S | S | S | S | I | R | I | S | R |
| CY46540 | R | S | S | S | S | R | R | S | R | S | S | S | S | R | I | R | S |
| CY46732 | R | S | I | S | R | S | R | I | R | S | R | S | R | R | S | R | S |
| CY46963 | R | S | S | S | R | S | S | S | R | S | I | S | S | R | S | R | S |
| CY46973 | I | S | S | S | I | S | S | S | S | S | S | S | S | S | S | S | S |
| CY47074 | I | S | S | S | S | S | S | S | S | S | S | S | S | S | S | S | S |
| CY47112 | S | S | S | S | S | S | S | S | S | S | S | S | S | S | S | S | S |
| CY47137 | S | S | S | S | S | S | S | S | R | S | S | S | S | R | S | I | S |
| CY47453 | S | S | S | S | S | S | S | S | S | S | S | S | S | S | S | S | S |
| CY47138 | I | S | S | S | S | S | S | S | S | S | S | S | S | S | S | S | S |
| CY48048 | R | S | S | S | S | S | S | S | R | S | S | S | S | R | S | R | S |
| CY48739 | S | S | S | S | S | S | S | S | S | S | S | S | S | R | S | S | S |
| CY49010 | R | S | S | S | S | S | I | S | S | S | S | S | R | S | R | S | S |
| CY49218 | R | S | R | S | S | S | S | I | S | S | S | S | R | R | R | S | R |
| CY49867 | I | S | S | S | S | S | S | S | S | S | S | S | S | S | S | S | S |
| CY49966 | R | S | S | S | S | S | S | S | R | I | S | S | S | R | S | I | S |
| CY50067 | S | S | S | S | S | S | S | S | S | S | S | S | S | S | S | S | S |
| CY50054 | S | S | S | S | S | S | S | S | S | S | S | S | S | S | S | S | S |
| CY50318 | I | S | S | S | S | S | S | S | S | S | S | S | S | S | S | S | S |
| CY51407 | S | S | S | S | S | S | S | S | S | S | S | S | S | S | S | S | S |
| CY52034 | R | S | S | S | I | S | S | I | S | S | S | S | S | R | R | S | S |
| CY52561 | I | S | S | S | S | S | S | S | S | S | S | S | S | S | S | S | S |
| CY52549 | S | S | S | S | S | S | S | S | S | S | S | S | S | S | S | S | S |
| CY52658 | R | S | R | I | S | S | R | S | S | S | S | S | I | S | R | S | I |
| CY52655 | S | S | S | S | S | S | S | S | S | S | S | S | S | S | S | S | S |
| CY52937 | I | S | S | S | I | S | S | S | S | S | S | S | S | S | S | S | S |
| CY53111 | I | S | S | S | S | S | S | S | S | S | S | S | S | S | S | S | S |
| CY53106 | I | S | S | S | S | S | S | S | S | S | S | S | S | S | S | S | S |
| CY53373 | I | S | S | S | S | S | S | S | S | I | S | S | S | S | S | S | S |
| CY53604 | R | S | S | S | S | S | S | I | S | S | S | S | S | R | S | S | S |
| CY53629 | I | S | S | S | S | S | S | S | S | I | S | S | S | S | S | S | S |
| CY53818 | R | S | S | S | R | S | S | S | R | S | R | S | S | R | S | I | S |
| CY53994 | R | S | S | S | S | S | R | I | S | S | S | S | I | S | S | S | S |
| CY54627 | I | S | S | S | S | S | S | S | S | S | S | S | S | R | S | S | S |
| CY54639 | R | S | R | I | R | R | R | R | S | S | R | S | R | S | R | S | R |
| CY54755 | I | S | S | S | S | S | S | S | S | S | S | S | S | S | S | S | S |
| CY54846 | R | S | S | S | S | S | S | S | S | S | S | S | S | S | S | S | S |
| CY54866 | I | S | S | S | S | S | S | S | S | I | S | S | S | S | S | S | S |
| CY54978 | I | S | S | S | S | S | S | S | S | S | S | S | I | S | R | S | S |
| CY55210 | I | S | S | S | S | S | S | S | S | S | S | S | S | S | S | S | S |
| CY55360 | I | S | S | S | S | S | S | S | S | I | S | S | S | S | S | S | S |
| CY55471 | R | S | S | S | R | S | S | S | R | S | I | S | S | R | S | R | S |
| CY56548 | I | S | S | S | S | S | S | S | S | S | S | S | S | S | S | S | S |
| CY56691 | R | S | S | S | S | S | S | S | S | S | S | S | I | S | R | S | S |
| CY56729 | S | S | S | S | S | S | S | S | S | S | S | S | S | S | S | S | S |
| CY56712 | R | S | S | S | S | S | S | S | S | I | S | S | I | S | S | S | S |
| CY57238 | R | S | R | S | R | S | R | R | I | I | R | S | R | R | R | S | R |
| CY57322 | R | S | R | S | R | S | R | S | S | S | I | S | I | S | R | S | S |
| CY57472 | S | S | S | S | S | S | S | S | S | S | S | S | S | S | S | S | S |
| CY57541 | R | S | S | S | S | S | I | I | S | S | S | S | I | S | S | S | S |
| CY57868 | S | S | S | S | R | S | S | S | S | I | I | S | S | S | S | S | S |
| CY58028 | I | S | S | S | R | S | S | S | S | S | I | S | S | S | S | S | S |
| CY58169 | R | S | S | I | S | S | S | S | S | S | S | S | S | S | S | S | S |
| CY58365 | R | S | S | S | S | S | S | S | S | S | S | S | S | S | S | S | S |
| CY58423 | R | S | S | S | R | S | S | S | S | S | R | S | S | S | S | S | S |
| CY58482 | R | S | S | S | S | S | S | S | I | S | S | S | S | R | S | S | S |
| CY58754 | R | S | R | S | S | S | S | I | S | I | S | S | R | S | R | S | I |
| CY58753 | R | S | S | S | R | S | S | S | R | S | I | S | S | R | R | S | S |
| CY59125 | I | S | R | S | S | S | S | S | S | S | S | S | I | S | R | S | I |
| CY59312 | R | S | S | S | S | S | S | S | S | S | S | S | S | S | S | S | S |
| CY59375 | I | S | S | S | R | S | S | S | R | S | R | S | S | R | S | I | S |
| CY59573 | I | S | S | S | S | S | S | S | S | S | S | S | S | S | S | S | S |
| CY59568 | I | S | S | S | S | S | S | S | S | S | S | S | S | S | S | S | S |
| CY59830 | S | S | S | S | S | S | S | S | S | S | S | S | S | S | S | S | S |
| CY60302 | R | S | R | S | S | S | I | I | S | S | I | S | R | R | R | S | S |
| CY60453 | R | S | R | S | R | S | S | I | S | S | I | S | S | R | R | S | S |
| CY60538 | R | S | S | S | R | S | R | S | R | S | I | S | I | R | R | I | S |
| CY60575 | R | S | S | S | R | S | S | S | S | S | R | S | S | S | I | S | S |
| CY60699 | R | S | R | S | R | S | I | I | S | I | I | S | R | R | R | S | R |
| CY60929 | R | S | R | S | S | S | R | R | S | I | S | S | R | S | R | S | R |
| CY60996 | R | R | I | S | R | S | R | I | R | S | R | S | R | R | I | R | S |
| CY61168 | R | S | R | S | R | S | S | I | S | I | I | S | I | S | I | S | I |
| CY61344 | R | S | S | S | R | S | S | S | S | I | I | S | S | R | S | S | S |
| CY61452 | R | S | S | S | R | S | S | S | I | S | I | S | S | S | S | S | S |
| CY61630 | I | S | S | S | S | S | S | S | S | I | S | S | S | S | S | S | S |
| CY61719 | I | S | S | S | S | S | S | S | S | S | S | S | S | S | S | S | S |
| CY62017 | R | S | S | S | S | S | S | S | S | S | S | S | S | S | S | S | S |
| CY62113 | R | S | S | S | S | S | S | S | S | I | S | S | S | S | S | S | S |
| CY62401 | R | S | S | S | R | S | S | S | R | I | R | S | S | R | S | R | S |
| CY62533 | R | S | S | S | R | S | S | S | R | I | I | S | S | R | S | R | S |
| CY62619 | I | S | S | S | R | S | S | S | R | S | R | S | S | R | S | I | S |
| CY62692 | I | S | S | S | R | S | S | S | I | S | I | S | S | S | S | I | S |
| CY62709 | R | S | S | S | R | S | S | S | S | S | I | S | S | S | S | S | S |
| CY62757 | I | S | S | I | S | S | S | S | S | S | S | S | S | S | S | S | S |
| CY62889 | R | S | S | S | R | S | S | S | I | S | R | S | S | R | S | I | S |
| CY63105 | R | S | S | S | R | S | S | S | R | S | I | S | S | R | S | S | S |
| CY63210 | R | S | R | S | R | S | R | I | S | S | R | S | R | S | R | S | R |
| CY63410 | R | S | S | S | S | S | R | S | S | S | S | S | I | S | R | S | S |
| CY63531 | S | S | S | S | S | S | S | S | S | S | S | S | S | S | S | S | S |
| CY63662 | R | S | S | S | R | S | S | S | R | S | R | S | S | R | S | R | S |
| CY63709 | R | S | S | S | S | S | S | S | S | S | S | S | S | S | S | S | S |
| CY63734 | R | S | S | S | R | S | I | S | S | I | R | S | I | R | R | I | S |
| CY63819 | R | R | I | I | R | S | R | I | R | I | R | S | R | R | S | R | S |
| CY64374 | R | S | R | S | S | S | R | I | S | S | S | S | R | S | R | S | R |
| CY64858 | R | S | S | S | R | S | S | S | S | R | I | S | S | S | I | S | S |
| CY65505 | R | S | S | S | S | S | S | S | S | S | S | S | S | S | S | S | S |
| CY65860 | R | S | I | S | R | S | S | S | S | I | I | S | I | S | I | S | I |
| CY65834 | R | S | S | S | R | S | S | S | I | S | I | S | S | S | S | S | S |
| CY66257 | R | S | S | S | R | S | S | S | I | I | I | S | S | S | S | I | S |
| CY66720 | R | S | S | S | S | S | S | S | S | S | S | S | S | S | S | S | S |
| CY67253 | I | S | S | S | S | S | S | S | S | S | S | S | S | S | S | S | S |
| CY67354 | R | S | S | S | S | S | S | S | S | S | S | S | S | S | S | S | S |
| CY67583 | R | S | S | S | R | S | S | S | R | S | I | S | S | R | S | R | S |
| CY67688 | R | S | S | S | R | S | S | S | S | S | I | S | S | R | S | S | S |
| CY67939 | R | S | S | S | S | S | S | S | S | S | S | S | S | S | S | S | S |
| CY68076 | S | S | S | S | R | S | R | S | S | S | R | S | R | R | S | S | S |
| CY68213 | R | S | S | S | R | S | S | S | S | S | S | S | S | R | S | S | S |
| CY68702 | R | S | S | S | S | S | S | S | S | S | S | S | S | S | S | S | S |
| CY68990 | I | S | S | S | R | S | S | S | R | I | I | S | S | R | S | I | S |
| CY69118 | R | S | I | S | R | S | S | I | R | R | I | S | R | R | I | R | R |
| CY69327 | I | S | S | S | R | S | S | S | R | S | S | S | S | R | S | S | S |
| CY70628 | R | S | I | S | R | S | I | I | S | S | I | S | R | S | R | R | R |
| CY70858 | I | S | S | S | S | S | S | S | S | S | S | S | S | S | S | S | S |
| CY70937 | R | S | S | S | R | S | S | S | R | S | S | S | S | R | S | R | S |
| CY70963 | R | S | S | S | S | S | S | S | S | S | S | S | S | S | S | S | S |
| CY71015 | R | S | S | S | R | S | S | S | S | S | S | S | S | S | S | S | S |
| CY71347 | I | S | S | S | R | S | S | S | S | R | I | S | S | S | S | S | S |
| CY72190 | R | S | S | S | S | S | S | S | S | S | S | S | S | S | S | S | S |
| CY72154 | I | S | S | S | S | S | S | S | S | S | S | S | S | S | S | S | S |
| CY72856 | R | S | S | S | S | S | S | S | S | S | S | S | S | S | S | S | S |
| CY74124 | R | S | S | S | R | S | S | S | S | I | S | S | S | S | S | S | S |
| CY74101 | R | S | S | S | S | S | S | S | S | I | S | S | S | S | S | S | S |
| CY74427 | R | S | R | S | S | S | I | I | S | R | S | S | R | R | R | S | R |
| CY74435 | R | S | S | S | S | S | S | S | S | I | S | S | S | S | S | S | S |
| CY74555 | R | S | R | R | R | I | R | R | S | I | R | S | R | R | R | I | R |
| CY74576 | I | S | S | S | R | S | S | S | S | I | S | S | S | R | S | S | S |
| CY74623 | R | S | S | S | R | S | S | S | S | I | S | S | S | S | S | S | S |
| CY74706 | R | S | S | S | S | S | S | S | S | I | S | S | S | R | S | S | S |
| CY74720 | I | S | S | S | S | S | S | S | S | S | S | S | S | S | S | S | S |
| CY74784 | R | S | S | S | S | S | S | S | S | I | S | S | S | S | S | S | S |
| CY74783 | S | S | S | S | S | S | S | S | S | S | S | S | S | S | S | S | S |
| CY74824 | R | S | S | S | R | S | S | S | S | I | S | S | S | R | S | S | S |
| CY74921 | R | S | S | S | R | S | S | S | S | R | I | S | S | S | S | S | S |
| CY75331 | S | S | S | S | S | S | S | S | S | I | S | S | S | R | S | S | S |
| CY75424 | I | S | S | S | S | S | S | S | S | R | S | S | S | S | S | S | S |
| CY75463 | I | S | S | S | S | S | S | S | S | R | S | S | S | S | S | S | S |
| CY75646 | R | S | S | S | S | S | S | S | S | R | S | S | S | S | S | S | S |
| CY75669 | S | S | S | S | S | S | S | S | S | R | S | S | S | S | S | S | S |
| CY75719 | R | S | S | S | R | S | S | S | S | S | I | S | S | S | S | S | S |
| CY75768 | R | S | S | S | S | S | I | S | R | R | S | S | R | R | R | R | S |
| CY75781 | R | S | S | S | S | S | S | S | S | R | S | S | S | S | S | S | S |
| CY75834 | I | S | S | S | R | S | S | S | R | R | S | S | S | R | S | R | S |
| CY75874 | R | S | S | S | R | S | S | S | R | R | S | S | S | R | S | R | S |
| CY76062 | R | S | S | R | R | R | R | I | R | R | R | R | R | R | R | R | R |
| CY76118 | R | S | S | S | S | S | S | S | S | I | S | S | S | S | S | S | S |
| CY76567 | I | S | S | S | R | S | S | S | S | R | R | S | S | R | S | S | S |
| CY76632 | I | S | S | S | S | S | S | S | S | R | S | S | S | S | S | S | S |
| CY77529 | S | S | S | S | R | S | R | S | R | S | S | S | I | R | S | I | S |
| CY77626 | R | S | S | S | R | S | S | S | S | I | S | S | S | S | S | S | S |
| CY78043 | R | S | S | S | R | S | S | S | R | R | S | S | S | R | S | S | S |
| CY78152 | R | S | S | S | R | S | S | S | S | I | R | S | S | R | S | S | S |
| CY78552 | R | S | S | S | R | S | S | S | S | S | R | S | S | S | S | S | S |
| CY78587 | R | S | S | I | S | S | I | S | R | I | S | S | R | R | R | R | S |
| CY78618 | R | S | S | S | R | S | S | S | S | I | S | S | S | S | S | S | S |
| CY78674 | R | S | S | S | R | S | S | S | S | I | R | S | S | R | S | S | S |
| CY78774 | R | S | S | S | R | S | S | S | S | S | S | S | S | S | S | S | S |
| CY78834 | R | S | S | S | R | S | S | S | R | S | R | S | S | R | S | I | S |
| CY79717 | R | S | S | S | R | S | I | S | R | R | R | S | I | R | R | I | S |
| CY80023 | R | S | S | S | R | S | S | S | R | R | R | S | S | R | S | I | S |
| CY80393 | R | S | S | I | R | S | S | S | R | S | I | S | S | R | S | R | S |
| CY80392 | R | S | S | S | S | S | S | S | S | S | S | S | S | S | S | S | S |
| CY80435 | R | S | S | I | R | S | S | S | R | R | I | S | S | R | S | R | S |
| CY80689 | R | S | S | S | R | S | S | S | S | I | R | S | S | S | S | S | S |
| CY80816 | R | S | S | S | S | S | S | S | S | S | S | S | I | S | R | S | S |
| CY101 | R | S | S | S | S | S | S | S | S | I | S | S | S | S | S | S | S |
| CY81202 | R | S | S | S | R | S | S | S | I | I | R | S | S | R | S | I | S |
| CY81369 | R | S | R | S | R | S | S | S | S | I | I | S | R | R | I | S | I |
| CY81409 | I | S | S | S | S | S | S | S | S | S | S | S | S | S | S | S | S |
| CY81392 | R | S | S | S | R | S | S | S | S | I | I | S | S | R | S | S | S |
| CY81574 | R | S | S | S | R | S | S | S | R | I | R | S | S | R | S | S | S |
| CY81737 | I | S | S | S | R | S | S | S | S | I | S | S | S | R | S | S | S |
| CY81755 | I | S | S | S | S | S | S | S | S | I | S | S | S | S | S | S | S |
| CY81785 | R | S | S | S | R | S | S | S | S | I | S | S | S | R | S | S | S |
| CY81906 | R | S | S | S | S | S | S | S | S | S | S | S | S | S | S | S | S |
| CY82048 | R | S | S | S | S | S | S | S | S | S | R | S | S | S | S | S | S |
| CY82274 | S | S | R | S | R | S | R | I | S | I | S | S | R | R | R | S | S |
| CY82446 | R | S | S | S | S | S | S | S | S | I | S | S | S | S | S | S | S |
| CY82661 | R | S | S | R | S | S | R | S | S | R | S | S | R | S | R | S | R |
| CY82629 | I | S | S | S | S | S | S | S | S | R | S | S | S | S | S | S | S |
| CY82687 | I | S | S | S | S | S | S | S | S | R | S | S | S | S | S | S | S |
| CY82705 | R | S | S | S | R | S | S | S | S | R | S | S | I | S | S | S | S |
| CY82899 | R | S | R | S | R | S | R | I | R | R | R | S | R | R | I | S | I |
| CY82953 | R | S | R | S | R | S | S | S | R | I | R | S | I | R | I | I | I |
| CY82994 | R | S | S | S | R | S | R | S | S | R | R | I | R | R | S | R | S |
| CY83137 | I | S | S | S | S | S | S | S | S | I | S | S | S | S | S | S | I |
| CY83235 | R | S | S | S | S | S | S | S | S | I | S | S | S | S | S | S | S |
| CY84998 | R | S | S | S | I | S | S | S | R | R | R | S | S | R | S | I | S |
| CY84999 | I | S | S | S | S | S | S | S | R | S | S | S | S | S | S | I | S |

AK amikacin; ATM: aztreonam; CAZ: ceftazidime; CIP; ciprofloxacin; CN; gentamicin; CRO: ceftriaxone; CTT; cefotetan; FEP: cefepime; IPM: imipenem; LEV: levofloxacin; MEM: meropenem; PRL: piperacillin; SAM: ampicillin/sulbactam; SCF: cefoperazone/sulbactam; SXT: trimethoprim sulfamethoxazole; TOB: tobramycin; TZP: piperacillin-tazobactam.

Table S2 Comparison of antibiotic resistance rates of *Morganella morganii* among different clinical settings in China.

| Antibiotics | Hospital ZY (n=82) (%) | Hospital SZY (n=112) (%) | Hospital NX (n=141) (%) | Hospital CY  (n=218) (%) |
| --- | --- | --- | --- | --- |
| CIP | 11.0 | 15.2 | 20.6 | 39.9 |
| SXT | 20.7 | 30.4 | 56.0 | 37.2 |
| CAZ | 8.5 | 9.8 | 34.0 | 19.3 |
| LEV | 14.6 | 12.5 | 2.1 | 15.6 |
| CRO | 6.1 | 9.8 | 22.0 | 12.8 |
| ATM | 1.2 | 8.9 | 2.8 | 12.4 |
| TOB | 3.7 | 5.4 | 6.4 | 10.1 |
| TZP | 0 | 5.4 | 5.7 | 8.7 |
| FEP | 0 | 2.7 | 0.7 | 1.8 |
| AK | 1.2 | 0 | 0 | 1.4 |
| MEM | 0 | 0 | 0 | 0.5 |

CIP, ciprofloxacin; SXT, trimethoprim-sulfamethoxazole; CAZ, ceftazidime; LEV levofloxacin; CRO, ceftriaxone; ATM, aztreonam; TOB, tobramycin; TZP, piperacillin-tazobactam; FEP, cefepime; AK, amikacin; MEM, meropenem. Hospital ZY: The First Affiliated Hospital of Sun Yat-sen University, Guangzhou, China; Hospital SZY: Guangdong Provincial Hospital of Traditional Chinese Medicine, Guangzhou, China; Hospital NX: General Hospital of Ningxia Medical University, Yinchuan, China. Hospital CY (this study): Beijing Chao-Yang Hospital of Capital Medical University, Beijing, China. The data of hospital ZY, hospital SZY, and hospital NX are retrieved from study by Guoxiu Xiang and colleagues (doi: 10.3389/fmicb.2021.744291).
